# Addressing the context and consequences of substance use, misuse, and dependence: A global imperative

**DOI:** 10.1371/journal.pmed.1003000

**Published:** 2019-11-26

**Authors:** Alexander C. Tsai, Margarita Alegría, Steffanie A. Strathdee

**Affiliations:** 1 Center for Global Health, Massachusetts General Hospital, Boston, Massachusetts, United States of America; 2 Harvard Medical School, Boston, Massachusetts, United States of America; 3 Mbarara University of Science and Technology, Mbarara, Uganda; 4 Disparities Research Unit, Department of Medicine, Massachusetts General Hospital, Boston, Massachusetts, United States of America; 5 Division of Infectious Diseases and Global Public Health, University of California at San Diego School of Medicine, San Diego, California, United States of America

## Abstract

In an Editorial, Guest Editors Alexander Tsai, Margarita Alegria and Steffanie Strathdee discuss the accompanying Special Issue on Substance Use, Misuse and Dependence.

Substance use, misuse, and dependence contribute immensely to the global burden of disease [1]. Their harms extend far beyond their corrosive effects on health, safety, and wellbeing and additionally include those associated with healthcare expenditures, productivity losses, criminal justice involvement, and other negative effects on social welfare [2]. The incidence and harms of substance use, misuse, and dependence involve multilevel explanatory factors [3,4]. Interventions to reduce their individual- and population-level harms can often be hampered by biological, psychological, and social complexity, especially because substance use is often syndemic with other health and social problems such as HIV infection, hepatitis B and C, mental health disorders, and violence [5,6]. It is in this context that *PLOS Medicine* devotes its November 2019 Special Issue to research on substance use, misuse, and dependence. The contributions to the issue cover a wide range of topics, including social determinants of substance use, health harms resulting from substance use, and interventions to prevent or reduce the harms associated with substance use. There are also a number of gaps in terms of the topics covered, the robustness of the evidence, and its global scope.

## Harms of opioid use

For the first time in several decades—and concomitant with the rise in opioid use, misuse, and dependence—life expectancy has declined in the United States [7], and life expectancy gains have stalled in Canada [8]. Consistent with these global estimates, in an accompanying paper for the Special Issue, Astrid Guttmann and colleagues [9] analyzed 2002–2016 national data from the United Kingdom and Canada to identify women who likely used opioids during pregnancy (proxied by an infant birth hospitalization record coded with neonatal abstinence syndrome) and demonstrated markedly elevated mortality rates over up to 10 years of follow-up. The elevated rates were particularly striking for mortality due to avoidable causes like unintentional and intentional injuries. Using 1998–2014 data from a large sample of primary care practices in the UK, John MacLeod and colleagues [10] show that coprescription with benzodiazepines was highly prevalent among patients receiving opioid agonist and partial agonist treatment and that coprescription was strongly associated with drug-related poisonings. This study adds to the relatively thin evidence base [11] about the potential hazards of benzodiazepine coprescription in the setting of opioid agonist treatment [12]. Although opioid agonist treatment should not be withheld from patients concurrently taking benzodiazepines or other central nervous system depressants, these studies suggest a need for vigilance by healthcare professionals providing care for such patients to minimize the risk of overdose or death [13]. Coprescription of alprazolam may warrant particularly heightened scrutiny, however, given that it is the short-acting benzodiazepine most frequently involved in drug overdose deaths [14].

## Determinants of opioid use–related harms

The elevated mortality risks facing people with opioid use disorders are attributable to a complex web of interrelated structural and psychological causes [15–18]. The concept of the “risk environment” [19] may be useful to reference here, given its focus on the interplay between various structural factors that increase vulnerability to morbidity and mortality. The study by Zehang Li and colleagues [20] provides an example of the use of spatiotemporal data to characterize one aspect of the risk environment. Applying a Bayesian space–time model to emergency medical services dispatch data on suspected heroin-related overdose incidents from Cincinnati in 2015–2019, the investigators identified significant spatial heterogeneity in the distribution of these calls, with strong associations with features of the built environment and temporal spikes corresponding to local media reporting.

Analyzing 2005–2016 claims data, Yu-Jung Wei and colleagues [21] identified more than 200,000 adults with new claims related to opioid use disorder or overdose. They found that, by the end of the study period, nearly one-half had filled no opioid prescriptions in the 12 months prior to an incident opioid use disorder diagnosis or overdose. Among those who had filled opioid prescriptions, nearly three-quarters were prescribed a mean daily dose lower than the threshold needed to trigger most risk stratification algorithms. Also noteworthy is the analysis of 2015–2016 data from the US National Survey on Drug Use and Health (NSDUH) by Joel Hudgins and colleagues [22]. These authors found that approximately 1 in 20 adolescents and young adults reported either past-year opioid use disorder or past-year nonmedical use of prescription opioids and that three-quarters of those reporting nonmedical use of prescription opioids had obtained them from outside the healthcare system. These estimates are generally consistent with trends identified in similar, previously published analyses of NSDUH data [23–26]. Thus, although opioid prescribing patterns undoubtedly played a significant role in how opioid use disorders came to be so highly prevalent [27–29] and asymmetrically distributed in the US [30–32], a public health response that focuses solely on prescribing behavior is likely to be ineffective in reducing the number of fatal and nonfatal opioid overdoses.

## Interventions to reduce the harms associated with opioid use

For people with existing opioid use disorders, opioid agonist treatment is known to reduce mortality [33,34]. Monica Malta and colleagues [35] add to this evidence base with a systematic review showing a wide range of health and prosocial benefits of opioid agonist treatment for people with opioid use disorders who are incarcerated or have recently been released. Opioid agonist treatment may have important collateral health effects as well. Analyzing data from a 3-country (i.e., Canada, the US, and Mexico) cohort of people who inject drugs [36–38], Charles Marks and colleagues [39] found that people who inject drugs and who receive opioid agonist treatment are approximately half as likely to assist others in initiating injection drug use. They then developed a deterministic, dynamic transmission model of initiation into injection drug use, ongoing drug use, and cessation of drug use. Currently about 1 in 5 people with opioid use disorders receive any kind of treatment [40]; if treatment coverage were doubled to 40%, Marks and colleagues’ model suggests that the number of initiations into injection drug use could fall significantly. This is an underappreciated benefit of opioid agonist treatment and underscores its potential public health impact, extending beyond its benefit to the individual.

To assist with prevention and treatment efforts, Jesse Yedinak and colleagues [41] aggregated data from 2015–2016 state and national data sources to estimate the proportions of Rhode Island residents in various states: at risk of, in treatment for, and in recovery from opioid use disorders. The authors’ cross-sectional approach requires assumptions about exchangeability in the transition probabilities of individuals at each stage and the absence of biases due to selective mortality, but their addition of the “recovery” stage is a novel modification to the existing framework. This body of work echoes the familiar “voltage drops” analogy pioneered by John Eisenberg and Elaine Power [42], who used this model to illustrate how the potential for high-quality care is lost at various stages of access, enrollment, and treatment. So-called treatment cascade models have been used to identify gaps in the access and treatment continuum for a wide range of health conditions, including HIV treatment [43], prevention of mother-to-child transmission of HIV [44], depression [45], and, most recently, opioid use disorders [46].

Finally, for people with opioid use disorders who either cannot or do not choose to achieve sustained remission, alternative approaches might be considered to reduce the harms associated with ongoing use. Stephanie Lake and colleagues [47] analyzed 2014–2017 data from Vancouver, Canada, on people who use drugs and who experience chronic persisting pain and found that daily cannabis use was associated with significant reductions in high-frequency nonmedical opioid use. This finding echoes previously published studies showing that expansions in access to marijuana in the US have been associated with reductions in opioid overdose mortality [48,49]. Among those for whom opioids remain the drugs of choice, use of supervised consumption facilities can reduce the risk of overdose mortality, and the potential for either individual-level adverse health effects or neighborhood-level adverse social effects appears to be minimal [50,51]. Mary Clare Kennedy and colleagues [52] contribute to this literature by showing that, among clients of the first supervised consumption facility in North America (in Vancouver), frequent utilization was associated with a reduction in all-cause mortality over 2006–2017. Although “deaths of despair” [53] ranked highly among the causes of death observed in this study, other nonaccidental causes of death (e.g., cancer, cardiovascular disease) were also prominent. In the US, only 1 supervised injection facility currently exists, although 13 cities have sought approval to support their implementation.

Ongoing misalignment between state and federal laws governing use of recreational marijuana and availability of supervised injection facilities seriously undermines harm-reduction efforts in the US. These and other interventions across multiple sectors involving healthcare, economic, and social welfare systems need to be scaled up, dramatically and immediately, in order to substantively reduce the number of opioid overdose deaths [54,55]. However, as discussed by Alexander Tsai and colleagues [56], the single most all-consuming force that restrains an effective policy and programmatic response to the opioid overdose crisis—through multilevel pathways that have nimbly adapted to the contours of the crisis over time—is the stigma attached to opioid use. Women whose children are affected by neonatal abstinence syndrome (studied by Guttmann and colleagues [9]) carry a stigma for the rest of their lives. Current media attention devoted to the “mommy drinking” myth (debunked in the Special Issue study by Sarah McKetta and Katherine Keyes [57]) is driven by the stigma resulting from the intersecting levels of scrutiny targeted toward women who parent and toward those who consume alcohol. Moreover, the disparate geospatial burden of opioid-related incidents, such as those studied by Zehang Li and colleagues [20], generates a stigma that attaches to entire neighborhoods [58]. Indeed, as a class, harm-reduction interventions have been tainted by stigma, leading to their chronic underfunding and underutilization. These and other forms of stigma must be eliminated before the overdose crisis can be successfully overcome. Tsai and colleagues [56] provide suggestions for anti-stigma interventions at multiple levels to achieve this goal.

## Gaps in the literature and the way forward

This issue of *PLOS Medicine* is notable for several gaps. First, the majority of contributions to the Special Issue concern the North American opioid overdose crisis, but the global burden of disease attributable to alcohol use disorders greatly exceeds that attributable to opioid use disorders [1]. Touching on harms owing to alcohol use, McKetta and Keyes [57] used data from the 2006–2018 US National Health Interview Survey to examine national trends in binge drinking and heavy drinking. Consistent with concurrently published findings from the NSDUH [59], they found that heavy drinking has declined or stabilized for most age/sex subgroups but that binge drinking has increased, particularly among women and older men.

A second evidence gap has to do with the global reach of the evidence. Although *PLOS Medicine* publishes research findings of general interest to the medical and public health communities, we received manuscript submissions describing research conducted in only a limited number of countries. In this Special Issue, the sole paper representing research conducted outside the US, Canada, and the UK is the report by Samantha Harris and colleagues [60], who analyzed Swedish register data from 1984–2016 to show that both refugee and nonrefugee migrants had lower rates of substance use disorders compared with Swedish-born individuals but that over time, the rates among migrants converged to that of Swedish-born individuals. The issue regrettably features no articles from Africa, Asia, or South America, and no articles focused on indigenous populations or on racial, ethnic, or sexual minority groups.

A third evidence gap has to do with the portfolio of methods underlying the evidence base. Causal methods have an important role to play in characterizing the relationships between exposures and outcomes when experimental data are difficult to come by (as one might expect in research on substance use disorders). Among the articles included in this Special Issue, only Kennedy and colleagues [52] used the “E-value” [61] to estimate the minimum strength of association on the risk ratio scale that an unobserved variable would need to have with both the exposure (frequent utilization of a supervised consumption facility) and outcome (all-cause mortality) to fully explain away their observed association. More studies using causal mediation analysis [62], instrumental variables [63], marginal structural models [64], natural experiments [49], regression discontinuity designs [65], and synthetic control methods [66] are needed. This Special Issue also lacks articles based on qualitative data. Qualitative methods can be used to study complex phenomena like substance use disorders in greater depth [67], probe for mechanistic pathways linking the phenomena of interest [68], and generate new insights that can be tested in future studies [69].

Collectively, the articles published in this issue highlight the scope of discovery and implementation needed to reduce the global burden of disease attributable to substance use, misuse, and dependence. Challenges—some related to science, others related to politics—are apparent. Multiple lines of evidence have already charted a road map that can be followed with respect to immediate policy making and program deployment. But there is a yawning chasm between what we know works to reduce the burden of disease from substance use disorders and what, at a societal level, is actually done about this burden. Eliminating this gap is not beyond our reach, given the available scientific evidence and substantial burden of ill health and suffering calling for prompt action, making it a public health imperative.
